# “Biological Geometry Perception”: Visual Discrimination of Eccentricity Is Related to Individual Motor Preferences

**DOI:** 10.1371/journal.pone.0015995

**Published:** 2011-01-19

**Authors:** Yannick Wamain, Jessica Tallet, Pier-Giorgio Zanone, Marieke Longcamp

**Affiliations:** 1 Laboratoire Adaptation Perceptivo-Motrice et Apprentissage, Université de Toulouse, Toulouse, France; 2 Institut de Neurosciences Cognitives de la Méditerranée, CNRS-Université de la Méditerranée, Marseille, France; The University of Western Ontario, Canada

## Abstract

**Background:**

In the continuum between a stroke and a circle including all possible ellipses, some eccentricities seem more “biologically preferred” than others by the motor system, probably because they imply less demanding coordination patterns. Based on the idea that biological motion perception relies on knowledge of the laws that govern the motor system, we investigated whether motorically preferential and non-preferential eccentricities are visually discriminated differently. In contrast with previous studies that were interested in the effect of kinematic/time features of movements on their visual perception, we focused on geometric/spatial features, and therefore used a static visual display.

**Methodology/Principal Findings:**

In a dual-task paradigm, participants visually discriminated 13 static ellipses of various eccentricities while performing a finger-thumb opposition sequence with either the dominant or the non-dominant hand. Our assumption was that because the movements used to trace ellipses are strongly lateralized, a motor task performed with the dominant hand should affect the simultaneous visual discrimination more strongly. We found that visual discrimination was not affected when the motor task was performed by the non-dominant hand. Conversely, it was impaired when the motor task was performed with the dominant hand, but only for the ellipses that we defined as preferred by the motor system, based on an assessment of individual preferences during an independent graphomotor task.

**Conclusions/Significance:**

Visual discrimination of ellipses depends on the state of the motor neural networks controlling the dominant hand, but only when their eccentricity is “biologically preferred”. Importantly, this effect emerges on the basis of a static display, suggesting that what we call “biological geometry”, i.e., geometric features resulting from preferential movements is relevant information for the visual processing of bidimensional shapes.

## Introduction

As established by many psychophysical studies, biological motion is a special stimulus for the visual system [1]–[3]. In a few hundred milliseconds, dynamic patterns of human actions can be recognized from extremely impoverished stimuli such as a few dots in motion [1]. A large number of studies have thoroughly investigated the various conditions in which this sensitivity is expressed [4]–[7].

A widely accepted explanation for this ability to recognize biological motion is that observers rely on their own knowledge about the laws that govern the motor system to process the visual stimulus. Notable instances are the Fitts' law [8], [9], the 2/3 power law [10]–[13], the effect of biomechanical constraints [2], [3], the isochrony principle [14], [15] and motor anticipation [16]–[17]. The phenomenon has been coined as « motor-perceptual interactions » [18]. At the brain level, the assumption that perception of biological motion relies on motor rules is supported by a stronger activation of motor brain regions, such as the premotor cortex, the inferior frontal gyrus, the supplementary motor area, the primary motor cortex and the cerebellum, when biological motion is observed, compared to non-biological motion or scrambled biological motion [19]–[21].

By definition, the study of biological motion perception requires the use of dynamical visual displays, because the features and laws of movements are highly related to time [1], [22]. However, some movements such as tracing, scribbling, or even reaching are also defined by their geometric features [23]–[26], and it can be hypothesized that motor-perceptual interactions are evoked not only by the time-varying features of movements, but also by their geometrical features, that is the shape of the path they form. In that case, motor-perceptual interactions should arise when only shape information is provided as a stimulus, isolated from the movement kinematics. The rationale is that shapes that resemble more what the motor system would spontaneously produce should have a special status for the visual system. But does the motor system display shapes preferences, and if so, how to identify them? In fact, spontaneous scribbling movements are characterized by the presence of strokes and elliptical trajectories that seem deeply rooted in the motor system because they can be observed in a variety of populations such as children as young as 2 years of age [27], normal adults [25], [28] and non-human primates [23], [24], [29], [30]. Ellipses (including their extremes exemplars, i.e., stroke and circle) therefore appear to be a compelling stimulus to test for motor-perceptual interactions selectively related to movement geometry.

Interestingly, several arguments indicate that within the whole range of ellipses, all eccentricities do not have the same value for the motor system. The drawings of young children, well described by Lurçat [27] are very informative in that sense: strokes and “elongated cycloids” (ellipses of intermediate eccentricity) are the first shapes to emerge in the drawing behavior, indicating the presence of “primitive” shapes in tracing movements. Although adults have learned how to control the production of various eccentricities, several studies show that some eccentricities, similar to the “primitives” observable in young children, are more resistant to the addition of constraints such as speed requirements [31]–[33] or cerebrovascular accidents [34], [35]. Such observations are interpreted as reflecting a greater stability of certain coordination patterns in the motor system [32], [33]. Experimentally, the accuracy and variability in the production of eccentricities ranging from a stroke to a circle has been systematically probed by Athènes et al. [36]. These authors showed that only the strokes and the ellipses with an intermediate eccentricity were reproduced with high accuracy and low variability, with a certain amount of variation between subjects. In addition, for most of the subjects, the circle was not accurately reproduced, but was rather transformed into an ellipse of low eccentricity.

**Figure 1:**
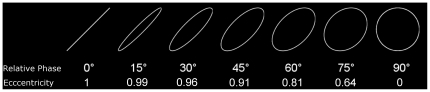
The 7 Standard Ellipses varying between 0° and 90° of relative phase (RP) by steps of 15° used in the discrimination task (with their respective eccentricity provided below the RP values).

In the framework of motor-perceptual interactions, we hypothesized that because some eccentricities are prevalent in the motor system, their visual discrimination might depend more on motor information. Several studies have also investigated visual processing of elliptical shapes [37]–[39], but today there is no reported sign that the visual discrimination of ellipses is influenced by geometrical features of motor preference in their production. To address this hypothesis, we used a dual-task paradigm [40]–[43]. A motor task was carried out in parallel to a visual discrimination task in which right-handers had to compare the eccentricities of ellipses and judge whether they differed or not by giving a response with their feet on pedals (see Fig. 1 for the stimuli used). The motor task aimed at mobilizing selectively the regions of the brain that are involved in hand movement programming and execution, in order to make them unavailable for the visual discrimination task. If the visual discrimination performance were dependent on the activity of such motor regions, it should be impacted by the motor task. This phenomenon should even be stronger for motor preferential ellipses. As a motor (possibly interfering) task, we chose a finger-thumb opposition sequence that is classically used by researchers who need to strongly and reliably activate the motor-related brain regions. [44]–[47]. In the visual discrimination task, we presented static ellipses in a single right-slanted orientation (Fig. 1) which is known to be produced most spontaneously by right-handers [32]. Based on previous studies in the field of motor control, we quantified eccentricity by means of the relative phase between two abstract orthogonal oscillators deemed to generate the trace [36], [48]. The relative phase (RP) corresponds to the time lag between the two oscillators, and informs both on the eccentricity and the orientation of ellipses. RP manipulation generates ellipses varying between right slanted stroke (ellipse with the maximal eccentricity) and circle (ellipse with no eccentricity), including ovals (ellipse with intermediary eccentricity) (see Fig. 1 for the correspondence between ellipses, eccentricity and RP). Thanks to this dual-task paradigm, we computed as a variable of interest the cost of the motor task on the discrimination sensitivity (d') relative to a control condition where the discrimination task was performed in isolation.

To assess the specificity of motor interference on visual discrimination, we manipulated two types of motor preferences. First, within the possible range of eccentricities between a stroke and a circle in the discrimination task, we considered separately for each participant the eccentricities that were motor preferential and those that were not, based on an assessment of individual spontaneous preferences in an independent graphomotor task. Second, we manipulated the hand that was performing the motor task. Given that spontaneous movements used to trace ellipses (and more generally for skills as drawing or writing) are quasi-exclusively performed with the dominant hand, we hypothesized that a motor task performed with the dominant hand would affect the simultaneous discrimination task more than a motor task performed with the non-dominant hand.

Overall, we report effects that are more specific than expected: the motor task impaired the discrimination sensitivity when it was performed with the dominant hand, but only when participants discriminated ellipses that we defined as preferential. When the motor task was performed with the non-dominant hand, and when the discriminated ellipses were non-preferential, the motor task had no significant cost on the discrimination sensitivity. This finding has two major implications: first, because we used a static display, it suggests that movement geometry is coded within the visual system without being mediated by kinematic variables, bringing the question of the neural mechanisms allowing its emergence; second it indicates that for a given individual, motor knowledge is relevant only for the visual processing of the shapes that would be the most spontaneously produced.

## Results

### Graphomotor Control task: assessment of individual spontaneous preferences in the production of ellipses

**Figure 2:**
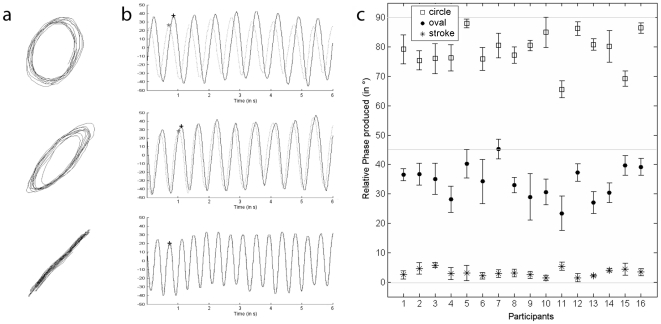
Results of the graphomotor task. (a) Example of the three shapes produced by one participant during one trial of the graphomotor task, where the instruction was to produce a stroke, an oval and a circle continuously for 10 s on a digitizing tablet. (b) Evolution of each oscillator x (solid line) and y (dotted line) as function of time. Relative Phase was computed as the time-lag between maxima (or minima) points (for example * and +) of each oscillator. (c) Individual results in the Graphomotor task: Relative Phase produced by each participant as a function of required shape (Group's means were 79.9, 34.1, and 3.2 respectively for circle, ellipse and stroke). A perfect circle has a RP of 90°, a perfect stroke has a RP of 0°, and an ellipse of intermediate eccentricity has a RP of 45° (see fig. 1).

This task aimed at identifying, for each participant, the eccentricities most spontaneously produced using an off-line analysis. It was systematically run after the dual tasks in order to avoid a possible influence of tracing the ellipses prior to the visual discrimination task. However, because the graphomotor task was used to analyze the discrimination task according to a distinction between motor preferential and non-preferential ellipses, we describe it before the dual tasks. Participants were required to draw series of strokes, ovals, and circles. Fig. 2c shows, for all the participants, the produced RPs as a function of the required shape. On average, the RP was around 3° for the stroke, 34° for the oval and 79° for the circle. As displayed in Fig. 2c, the produced RPs varied from participant to participant, especially for the oval.

### Dual-Tasks: interactions between activation of the motor system and visual processing of ellipses

#### 1. Visual Task: Discrimination of eccentricity

**Figure 3:**
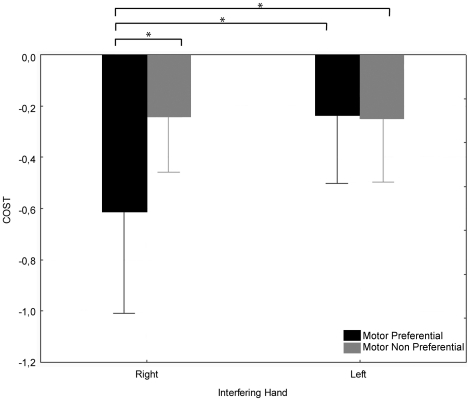
Cost of the motor interfering task as a function of the interfering Hand (Right or Left) for the two categories of ellipses (Motor Preferential or Motor Non-preferential). Error bars represent the 95% confidence intervals.

Table 1 presents the mean values of the discrimination sensitivity (*d'*, computed according to the Signal Detection Theory [49]), separately for Motor Preferential and Non-preferential ellipses during the three conditions of the interfering task (control: without interfering motor task, left hand and right hand). Fig. 3 shows the cost of the motor interfering task (difference between d' in the interfering condition and *d'* in the control condition) as a function of the Interfering hand (Right vs. Left) and of Motor Preference (Preferential vs. Non-preferential). The cost quantifies the impact of moving each Interfering Hand (Left and Right) on visual discrimination of Motor Preferential and Motor Non-preferential ellipses. When its value differs from 0, it indicates that the hand movement impacts visual discrimination. We therefore first tested whether the cost was significantly different from 0, using a t-test for each combination of the two independent variables (Interfering Hand and Motor Preference). Since 4 t-tests were performed, we used the Bonferroni procedure to cope with the problem of multiple comparisons. The significance threshold was set at *p*<0.0125. In the right Interfering Hand condition, the Cost differed significantly from 0 for Motor Preferential ellipses (t_15_  = 3.28, *p*<0.005). It did not significantly differ from 0 for the other 3 conditions (t_15_  = 2.35 for Motor Non-preferential ellipses with Right Interfering Hand, t_15_  = 1.87 for Motor Preferential ellipses with Left Interfering Hand and t_15_  = 2.17 for Motor Non-preferential ellipses with Left Interfering Hand).

**Table 1:** discrimination sensitivity (d').

d'		Motor Preference		
		Preferential		Non-Preferential
		All preferential	Without stroke	
Condition of the Interfering Task	Right	2.21 (0.73)	1.21 (0.62)	1.68 (0.63)
	Left	2.58 (0.46)	1.54 (0.41)	1.67 (0.5)
	Control	2.70 (0.64)	1.67 (0.51)	1.92 (0.44)

Mean (Standard deviation) values of the discrimination sensitivity (d') for motor preferential and non preferential ellipses during the three conditions of the Interfering hand task (Control, Right and Left hand). For Motor Preferential shapes, average d' values are presented both including values for the stroke and without values for the stroke.

A 2×2 repeated measures ANOVA with the cost as the dependent variable revealed a significant interaction between Interfering Hand and Motor Preference [F(1, 15)  = 8.20, *p*<0.012, η^2^_p_  = 0.35]. Post-hoc comparisons showed that the cost for the Motor Preferential ellipses in the Right Interfering Hand condition differed significantly from the 3 other conditions (Motor Non-preferential ellipses with Right Interfering Hand, *p*<0.013; Motor Preferential ellipses with Left Interfering Hand, *p*<0.012; Motor Non-preferential ellipses with Left Interfering Hand, *p*<0.016), which did not differ significantly from each other).

Table 1 suggests important differences in the *d'* of Preferential and Non-preferential ellipses. In fact, the apparent higher sensitivity for preferential ellipses is exclusively due to the inclusion of the stroke among the Preferential ellipses. Motorically, the stroke is clearly a Preferential ellipse of extreme eccentricity, but its perceptual status is particular because the distinction between the eccentricity of the stroke and of the ellipse of closest eccentricity is very easily perceptible. To check that the observed effects were not due to the inclusion of the stroke among the Preferential ellipses, we ran the same 2×2 repeated measures ANOVA without including the stroke in the calculations (d' values and figure with the cost are given as supplementary material). Despite decreased power consequent to the inclusion of less measurements in the mean cost for preferential ellipses, we still observed a significant interaction between Interfering hand and Motor Preference [F(1, 15)  = 5.05, *p*<0.041, η^2^_p_  = 0.25].

#### 2. Motor task: finger-thumb opposition sequence

The number of finger-thumb oppositions was counted in the time-window of each discrimination trial, in order to monitor possible variations of the rate of the motor sequence according to the type of visual stimuli, and the interfering hand. An ANOVA revealed neither significant main effect nor interaction.

## Discussion

Classically, a dual-task paradigm is used to test, at a behavioral level, if neural resources called upon by a concurrent task participate to a principal task [40]–[43]. In order to investigate whether motor-perceptual interactions occur during visual discrimination of static ellipses, we used a finger-thumb opposition sequence which is well known to selectively and strongly activate the motor neural networks [44]–[47]. We tested whether the cost of this motor task on the discrimination performance depended on the laterality of the hand mobilized and whether it differed between “motorically” preferred and non-preferred ellipses.

First, the cost of the finger-thumb opposition sequence significantly differed from zero for motor preferential ellipses when the sequence was performed with the right hand. Second, this cost depended on an interaction between the hand mobilized in the motor task and the motor preference for certain ellipses. It was larger when motor preferential ellipses were discriminated while the motor task was performed with the right hand than in the other 3 conditions. Performance in the finger-thumb opposition sequence was controlled by the experimental procedure and actually did not display any difference between hands that could explain these results.

To our knowledge, this finding provides the first demonstration of interactions between low-level motor preferences and visual discrimination of stimuli presented on a static form. In fact, some studies showed that motor-perceptual interactions are induced by visual perception of static stimuli, such as face expressions [50], static snapshots of dynamic actions [1], [2], [51], pictures of interactions between hands and tools [52], or symbols and letters [53]–[59]. In comparison with these studies, our stimuli did not represent or even allude to actions from which a meaning or an intention could be directly inferred, but much lower-level parts or “primitives” of movements [24], [28], with some exemplars being easier to produce than others [32], [36]. Our results suggest that such movement geometric primitives, presently identified as the individual's preferences in a graphomotor task, give to some ellipses a particular status for visual discrimination.

The selectivity of the effect for motor preferential ellipses precludes explanations in terms of both attentional demands and stimulus - response compatibility effects: first, if attentional demands were to impact the discrimination performance, this impact should be the same for preferential and non-preferential ellipses and should also be larger when the motor task is performed with the left hand, as movements of the non-dominant hand are deemed to be more demanding [60]. Second, because the ellipses were presented in a single (right-slanted) direction, it is possible that their processing is more dependent on the mobilization of the right hand [61], revealed for instance that right-hand key-presses interfered more with responses to a right-pointing than to a left-pointing arrow). However, the interaction, with a selective impairment of visual discrimination for motor preferential ellipses when participants mobilized their dominant hand, makes an interpretation in terms of a simple interference between the direction of presented ellipses and the hand mobilized unlikely.

A challenging explanation of our results is that performance in ellipses discrimination is dependent on the solicitation of motor neural networks for motor preferential ellipses, possibly because motor preferences, which are more precise in this case and which are linked to the right hand movements, provide additional information for their visual discrimination. The impact of motor preferences in visual perception has already been demonstrated in several psychophysical and neuroimaging studies that used dynamic point-light displays [4]-[17], [62]. These studies established that the laws of human movement influence perceptual judgments. The present results extend these findings by showing that preferences for certain eccentricities impact the visual processing of ellipses: the closer the eccentricity of the visual stimulus is to what the motor system would produce spontaneously, the greater the participation of the motor system to its visual processing. This suggestion is also in line with studies that compared individuals with different degrees of motor expertise and showed that the activation of motor regions during observation of motor skills is directly correlated with the observer's expertise [19], [63]–[66]. In addition, recent psychophysical studies clearly demonstrated that motor preferences in typewriting affected both the “likeability” [67] and the recognition memory [68] of visually presented letter pairs. Interestingly, these effects were shown to be cancelled when a concurrent finger tapping task was performed.

Interestingly, human movement laws are not the only type of information observers can rely upon when processing visual motion. Earlier studies have demonstrated that information such as weight, elasticity, length… can be accurately retrieved from kinematic displays of physical phenomena (free fall, pendulum motion, weight lifting… [69]–[71]) proving that observers are sensitive to more general laws of physics. It would be of interest to check whether visual processing of static displays embedding information about these laws of physics (for instance the display of the trajectory of a falling object) also implicitly take into account these laws. Furthermore, it is possible that knowledge related to biological motion and laws of physics interact in the processing of static displays when the relevant information is present.

Our results add to the growing body of literature on the impact of the current state of the motor system on visual perception [72]. For instance, when participants are engaged in actions like carrying a weight, walking, drawing, or acting upon objects, performances in visual perceptual tasks are biased [73]–[77]. Such a bias of perceptual performance while a motor concurrent task is achieved supports the idea that the two tasks recruit partly overlapping motor neural networks. Here, we chose a motor task that was quite unspecific, and aimed at broadly activating the neural networks supporting hand movements, rather than specifically targeting graphic movements. It is likely that actual graphic movements rely only on a subset of the regions involved in the finger-thumb opposition task, and perhaps also depend on the activation of extra regions. This limits possible conclusions in terms of specific neural bases of the interference. Despite this limit, the motor task has the advantage not to share any other process with the discrimination task than the presumed involvement of low-level representations of hand movements.

In summary, the observed stronger interference of a motor task performed with the right hand on visual discrimination of motor preferential ellipses strongly suggests that visual discrimination of ellipses depends on the state of the motor neural networks controlling the dominant hand, but only when their eccentricity is biologically preferred. In addition to previous results showing that kinematic/time features impact on visual perception of biological motion, our results suggest that “biological geometry”, that is the geometric/spatial features resulting from preferential movements is relevant information for visual processing of static bidimensional shapes. This new finding brings two interesting perspectives: first, can “biological geometry” give rise to activations in the brain regions controlling the movements of the hand, similar to biological motion [19]–[21]? Even if this question calls for further neuroimaging investigations, the answer is likely to be positive since when these networks are made unavailable, the discrimination sensitivity is impaired. Second, is biologically preferred geometry processed differently within the visual system? It is for instance possible that, through an impulse from the motor system, the static visual information is transformed into “dynamic mental representations” [78] and therefore processed by motion sensitive visual areas MT and STS [79], [80]? Alternatively, the behavioral effect we report in the present study might not be related to differential patterns of activity in the visual system, but to the direct participation of hand-related motor regions to the discrimination processes. Further investigations are needed to clarify the interplay between visual and motor regions during the discrimination of preferential vs. non-preferential shapes. Finally, it should be noted that the effect we evidenced concerns bidimensional shapes. The motor-perceptual processes at play when 3-D objects have to be discriminated in the environment are likely to be different and more related to grasping mechanisms [81]. Nonetheless, the implications of our finding remain important, because in modern societies a great amount of information is transmitted and gathered “bidimensionally” through books, paintings, and artwork and texts displayed in computer screens or other bidimensional media.

## Materials and Methods

### Ethics Statement

The experimental procedures were approved by the ethics committee of the Paul Sabatier University in accordance with the Helsinki declaration. All participants signed an informed consent form prior to their participation.

### Participants

Sixteen unpaid participants (7 males and 9 females, mean age  = 24.25; SD  = 3.95) participated in the experiment. All were right-handers according to the Edinburgh Handedness Inventory (mean  = 88,07; SD  = 13.4 according to [82]). All were naive to the purpose of the experiment and presented no impairment impeding the visual perception or the motor production of the stimuli. None of the participants was experienced in writing with the left hand or practiced physical or musical activities implying the hands.

### Material

Participants were seated at a distance of 114 cm in front of the computer screen (1280×1024, 100 Hz) in a dimly illuminated room.

Twenty five ellipses were generated with Matlab©, according to two parametrical equations simulating the evolution of two orthogonal oscillators, x(t) and y(t):
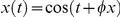


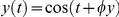



where 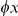 and 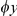 are the phases of each oscillator. By this mean, the ellipses were described in terms of relative phase (RP), which refers to the difference between the phases of the two oscillators [36], [48].
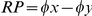



**Figure 4:**
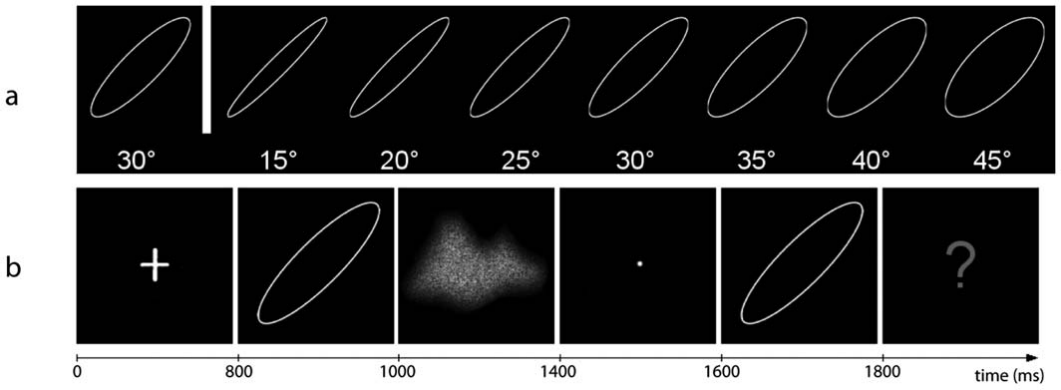
Organization of the Visual discrimination task. (a) Example of the relationship between one Standard Ellipse (here 30°, left side of the figure) and its corresponding 7 Test Ellipses. (b) Time sequence of one experimental trial.

The matlab code used to generate the ellipses is provided as supplementary material (Text S1). The ellipses were white on a black background, measured approximatively 2 cm (foveal vision, 1° visual angle) and were classified into two categories: standard ellipses (N = 7) which RP varied between 0° and 90° by steps of 15° (Fig. 1), and test ellipses (N = 7 for each standard ellipse) which were either identical to the standard ellipse, or differed in their RP from standard ellipses between −15° and 15° by steps of 5°. For example, the standard ellipse 30° was associated with 7 test ellipses: the ellipse 30° itself and six other ellipses of which RP were ranged between 15° to 45° by 5° steps (15°, 20°, 25°, 30°, 35°,40°, 45°; Fig. 4a.). Note that the stroke (0°) was only associated to 4 test ellipses (0°, 5°, 10°, 15°) because the negative RP values (−5°, −10°, −15°) leads to the same eccentricities.

Two response switches were placed under participants' feet. A force sensor (I.E.E. FSR 174) was placed on the last phalanx of each finger except the thumb, in order to control the performance of the finger-thumb opposition sequence. The switches and sensors were connected to an analogic acquisition card (NI PCI-6503) which allowed a temporal precision of 1 ms.

### Procedure

Each participant was first submitted to the dual-tasks: the visual ellipse discrimination tasks and the motor finger-thumb opposition continuous sequence, which were performed simultaneously. No priority was given to either task.

After the dual tasks, participants had to perform a graphomotor task

#### 1. Graphomotor Control Task

One trial consisted in tracing three shapes, a circle, an oval and a stroke, oriented at 1∶30 on a digitizing tablet (Wacom Intuos 3 a3). The order in which the shapes had to be produced was indicated on a card (C  =  circle, E  =  oval, T  =  stroke or “trait” in French) placed on the top of the tablet, and each shape had to be traced continuously for 10 s. A beep (after 10 s and 20 s) indicated to switch to the second and the third shape. Participants had to maintain the stylus in contact with the digitizing tablet during the whole trial. Six trials were performed, according to a counter-balanced order. No feedback of the trace was given on the digitizing tablet.

#### 2. Dual tasks

In the discrimination task, participants had to compare, in each trial, a standard ellipse with a test ellipse and to judge whether they were identical or different. One experimental trial lasted for 3200 ms and was composed of five successive events: a fixation mark (800ms), a standard ellipse (200ms), a mask (400ms), a second fixation mark (200ms), and the test ellipse (200ms). A red question mark (1400ms) signalled the end of the trial (Fig. 4b). Since the finger-thumb opposition sequence was performed simultaneously with one hand, participants had to respond with their feet by depressing response switches. The association between the foot (right or left) and the assignment of the response (identical or different) was counterbalanced across participants and trial order was randomized.

The discrimination task was composed of six blocks of 180 trials: two in each condition of the finger-thumb opposition sequence (control, left hand, right hand). Given that each standard ellipse could be compared to either itself or a test ellipse, we administrated 2 trials for each pair of different ellipses and 12 trials for pairs of identical ellipses, except for the stroke (0°).The order of the conditions in the discrimination task was counterbalanced across participants, and each block took about 10 min.

During the whole duration of the discrimination task, participants had to perform a finger-thumb opposition task where they had to sequentially press their thumb against the last phalanx of each finger (respectively index, medius, annular and auricular, then in the reverse order) at a spontaneous but constant cadence. The finger-thumb opposition sequence was performed either with the right or with the left hand. The control condition was realized without any finger-thumb opposition sequence. The three conditions were trained in familiarization blocks before the start of the discrimination session. In each block, participants started performing the finger-thumb opposition sequence. When a comfortable cadence was reached, the discrimination task started.

### Data processing

#### 1. Graphomotor Control Task

The first and last two seconds of each sequence were rejected in order to withdraw the transition between the produced patterns. When ellipses are traced continuously, the variations along the x- and y-axes are sinusoids (Fig. 2b). A cycle-by-cycle point-estimated RP was calculated (see [83], for details) and averaged for each pattern (Circle, Oval and Stroke, across 6 trials, see Fig. 2a). We considered these “spontaneous” average RPs to be preferential, allowing us to split, for each participant, the visual stimuli in the discrimination task into two categories of Motor Preference: Preferential vs. Non-preferential. Among the 7 ellipses used as standard in the discrimination task (see Fig. 1), we considered those 3 ellipses with RPs closest to the individual spontaneously produced average RPs for the required stroke, oval and circle as Preferential. For instance, for the participant whose data in the graphomotor task are depicted in Fig. 2a and b, the spontaneously produced average RPs were 79°, 36° and 2°. In the discrimination task, the closest standard ellipses were thus 75°, 30° and 0°. These were classified as preferential and the remaining standard ellipses 15°, 45°, 60° and 90° were classified as non-preferential. The same rationale was applied for each participant. For one of the participants, the spontaneously produced RP for the oval was 37.3° and thus fell exactly in between the standard ellipses 30° and 45°. We therefore took the average d' values for the discrimination trials involving standard ellipses of 30° and 45° as preferential for that participant.

#### 2. Dual-tasks

For the discrimination task, an index of discrimination *d'* was computed, according to Signal Detection Theory, for each condition (control, left, right) and each (7) Standard Ellipse [49]. We evaluated the cost of performing the finger-thumb opposition sequence simultaneously by subtracting *d'* in the control condition (without interfering task) to *d'* with interfering task in each finger-thumb opposition condition (left and right). This computation was carried out separately for Motor Preferential and Motor Non-preferential ellipses. A trial was considered preferential when the standard ellipse (i.e., the ellipse presented first, see Fig. 4) had been classified as preferential. Two subjects displayed clearly deviant values of the cost in one of the conditions (subject 9 for the right hand/Non-preferential ellipses and subject 15 for the right hand/Preferential ellipses, value above the group mean + 2SDs). We replaced those two deviant values by the group mean in the corresponding condition.

In addition, a 2 (Interfering Hand) ×2 (Motor Preference) repeated measures ANOVA was carried out with the cost as the dependent variable. Post-hoc comparisons (Scheffé's test) were used to test between single levels of the independent variables. For these analyses, we set the significance threshold at *p*<.05 and we report the partial eta squared values (η^2^_p_) as a measure of the goodness of fit of the model.

For the Finger-thumb opposition sequence, we counted the number of presses occurring in the time-window of each discrimination trial (between the onset of the standard ellipse and the response signal). This allowed a repeated measures 2 (Interfering Hand) ×2 (Motor Preference) ANOVA on the number of presses as the dependent variable. Because the opposition required a greater effort to press the ring and the little fingers against the thumb, some of the oppositions were missed during the task because the sensors were not correctly pressed. In order to avoid any possible bias due to this measurement problem, we quantified the rate of the finger thumb opposition sequence by counting only the number of presses between the thumb and the index.

## Supporting Information

Text S1 **Matlab code used to generate the ellipses used as stimuli in the discrimination task.**
(DOC)
