# Supplementary material for: “Biological Geometry Perception”: Visual Discrimination of Eccentricity Is Related to Individual Motor Preferences
Source: PLoS One. 2011 Jan 19;6(1):e15995. doi: 10.1371/journal.pone.0015995 (PMC3023766; doi:10.1371/journal.pone.0015995)
Supplement: Text S1 — Matlab code used to generate the ellipses used as stimuli in the discrimination task. (DOC) [file pone.0015995.s001.doc]

t = [-pi:0.0001:pi];

osci1= cos(t);

rpp=['000';'005';'010';'015';'020';'025';'030';'035';'040';'045';'050';'055';'060';'065';'070';'075';'080';'085';'090';'095';'100';'105'];

k=0;

for i=1:22

figure (i);

figure('Units','pixels', ...

'Color',[0 0 0], ...

'Name',rpp(k+1,:), ...

'MenuBar','figure', ...

'NumberTitle','off', ...

'Position',[500 500 118 80], ...

'Resize','off');

set(gcf,'Color',[0,0,0]);

osci2 = cos(t+k*(pi/36));

k=k+1;

subplot(1,1,1); plot(osci1,osci2,'Color',[0.40, 0.40, 0.40],'LineWidth',1)

set(gca,'Color',[1, 1, 1]);

axis ([-1.2 1.2 -1.2 1.2]);

axis equal

axis off

set(gcf, 'PaperPositionMode', 'manual');

end;
